# A Refractory, Gas-Predominant Subgroup of Irritable Bowel Syndrome Markedly Improved With Relatively Long-Term Paroxetine Treatment: A Preliminary Study

**DOI:** 10.7759/cureus.85494

**Published:** 2025-06-07

**Authors:** Kazunori Mine, Yusuke Murata, Minoru Fujino, Takehiko Fujino, Munechika Enjoji

**Affiliations:** 1 Faculty of Psychosomatic Medicine, BOOCS Clinic Fukuoka, Fukuoka, JPN; 2 Department of Pharmacotherapeutics, Faculty of Pharmaceutical Sciences, Fukuoka University, Fukuoka, JPN; 3 Faculty of Internal Medicine, BOOCS Clinic Fukuoka, Fukuoka, JPN

**Keywords:** amitriptyline, flatulence, functional gastrointestinal disorder, gas symptom, irritable bowel syndrome, paroxetine, pharmacotherapy

## Abstract

Background

The intestinal-gas symptoms of irritable bowel syndrome (IBS) are particularly bothersome and refractory. In our Japanese clinical practice, we often experience IBS patients with severe complaints of excess ‘flatulence’ or ‘rumbling’ as well as bloating, and have considerable difficulty in treating them. However, few studies have been reported on this subgroup of IBS for whom intestinal-gas symptom is the most bothersome *(gas-predominant IBS*).

Aims

The aim of this study was to characterize the features of refractory *gas-predominant IBS* and to find an effective treatment strategy.

Methods

One hundred and forty-six consecutive patients who fulfilled the Rome IV criteria for IBS were divided into subgroups according to the most distressing symptom. Among these subgroups, we focused on a *gas-predominant IBS* group. ‘Flatulence’ is defined as ‘excess flatulence’ or ‘incontinence of flatulence’. Patients with *gas-predominant IBS* were treated with paroxetine, amitriptyline, or both. ‘Remission-like improvement’ was defined as achieving a severity rating of 30% or less for all IBS symptoms within 24 months, and no relapse occurred for at least six months.

Results

Of the 146 patients, 31 (21.2%) were classified ashaving* gas-predominant IBS. *All were refractory to conventional treatment. Ten did not meet the required treatment conditions (lack of treatment-related information (n=6) and deviation from the treatment protocol (n=4)) and were excluded, leaving the data of 21 patients available for study. Of the 21 studied patients, the most bothersome symptoms were ‘flatulence’ in 14 (66.7%), ‘rumbling’ in three (14.3%), both ‘flatulence’ and ‘rumbling’ in three (14.3%) and bloating in one (4.8%). Notably, 17 (81.0%) rated ‘flatulence’ and six (28.6%) rated ‘rumbling’ as one of the worst symptoms. Fifteen (71.4%) of the 21 patients exhibited very high anxiety scores for both state and trait anxiety, while a total of two (9.5%) were diagnosed with a psychiatric disorder, major depressive disorder. Eighteen (85.7%) of the 21 patients achieved ‘remission-like improvement’ with five to 23 months of treatment with paroxetine (n=14), amitriptyline (n=2), or both (n=2), and no relapse occurred for at least six months.

Conclusions

In Japan, there is a distinct refractory subgroup of IBS who complain of intestinal-gas symptoms such as ‘flatulence’ or ‘rumbling’ as their most distressing symptom. Further studies are needed on the prevalence and regional specificity of *gas-predominant IBS* in Japan and globally. Paroxetine treatment for a relatively long period may be effective in treating refractory *gas-predominant IBS*. However, this study was open-label and lacked a control group, and future investigations are needed to determine the most appropriate use of paroxetine for the treatment of *gas-predominant IBS.*

## Introduction

Irritable bowel syndrome (IBS) is a widely prevalent functional gastrointestinal disorder. Based on the Rome IV criteria, IBS is characterized by recurrent abdominal pain associated with defecation and with alterations in stool frequency or form [1]. Because IBS is not a single disease entity, but rather consists of several different disease states, a patient with IBS is treated based on the patient’s most bothersome symptoms. In general, the treatment strategy targets abdominal pain, diarrhea, constipation, and/or intestinal-gas symptoms. The Rome IV criteria classify it into three predominant subtypes: IBS with constipation, IBS with diarrhea, and mixed IBS. Because the classification into IBS subtypes is based on the patients’ perception of their predominant type of abnormal stool consistency, IBS with intestinal-gas as the predominant symptom is not included as a Rome IV IBS subtype. However, intestinal-gas symptoms such as abdominal bloating have a high prevalence and can markedly impair the quality of life of affected IBS patients [2,3]. Bloating is “a subjective sensation in any abdominal region experienced by patients as fullness, swelling, trapped gas or gaseousness, or tightness” [3]. In addition, intestinal-gas symptoms are considered to be particularly bothersome for IBS patients [4,5]. It is also recognized that the intestinal-gas symptoms of IBS are extremely difficult to treat and that the majority of medications designed for this indication have not been helpful [6]. To date, a small number of studies have reported on a subgroup of IBS for which the most bothersome symptom is intestinal-gas [3,7,8].

In Japanese clinical practice, we often experience IBS patients with severe complaints of excess ‘flatulence’ or ‘rumbling’ and have considerable difficulty in treating them. However, few studies have focused on the ‘flatulence’ and ‘rumbling’ of patients with IBS [5,9]. Malagelada et al. [10] defined ‘flatulence’ as the evacuation of large amounts of gas through the anus. We have essentially adopted this definition, with ‘flatulence’ in this paper clinically defined as excessive flatulence and incontinence of flatulence. Previous studies did not strictly define flatulence as a medical term, often referring to it as wind, flatus, or gas, etc., and the term flatulence was sometimes seen as interchangeable with bloating. This means that few studies have been done specifically on ‘flatulence’ as a symptom of IBS and indicates the difficulty of studying this topic.

From the above, we hypothesized that there exists a distinct intestinal-gas symptom predominant subgroup of IBS *(gas-predominant IBS*) for which the most distressing symptom is ‘flatulence’ or ‘rumbling’, as differentiated from bloating, and that it is refractory. The aim of this study was to confirm the existence of and to characterize the clinical features of *gas-predominant IBS* and to develop a more effective strategy for the treatment of patients with *gas-predominant IBS* suffering from ‘flatulence’ or ‘rumbling’, as well as bloating.

## Materials and methods

Subjects

The study participants were taken from 146 consecutive outpatients with IBS who first visited the Faculty of Psychosomatic Medicine, BOOCS Clinic in Fukuoka City, Japan, during the period from January 2018 to March 2023 and who met the enrollment criteria. They were diagnosed by a doctor specializing in gastroenterology as having IBS, but did not have any significant physical illness; all patients fulfilled the Rome IV criteria for IBS [1]. They were also given psychiatric and psychosomatic assessments by a doctor specializing in psychosomatic medicine.

In this study, ‘flatulence’ as an intestinal-gas symptom is strictly defined as ‘excess flatulence expelled through the anus’ or ‘incontinence of flatulence’, and ‘rumbling’ is defined as a rumbling in the bowel or borborygmus. After being explained the above definitions, the patients were asked, “Which symptom among abdominal pain, diarrhea, constipation, bloating, ‘flatulence’, or ‘rumbling’ has bothered you the most?”

Patients were then divided into subgroups according to the most distressing intestinal symptoms. The subgroups were as follows: *gas-predominant IBS*, *pain-predominant IBS*, *diarrhea-predominant IBS*, *constipation-predominant IBS*, *pain and diarrhea-predominant IBS*, and *pain and constipation-predominant IBS*. The present study focused only on the group classified as *gas-predominant IBS*.

IBS severity scoring of patients in the *gas-predominant IBS* group

The severity of the IBS of each *gas-predominant IBS* patient was assessed using the IBS Symptoms Severity Scale questionnaire (IBS-SSS) [11], and the patients were asked to rank bloating, ‘flatulence’, and ‘rumbling’ in their order of severity.

Psychiatric assessment

All subjects had a psychiatric assessment. The DSM-V criteria (The fifth edition of the Diagnostic and Statistical Manual of Mental Disorders, American Psychiatric Association, 2013) were used for the diagnosis of comorbid psychiatric disorders. The Japanese version of the Spielberger State-Trait Anxiety Inventory (STAI) was used to assess the anxiety level [12].

In the case of a patient diagnosed as having a comorbid depressive disorder, a Japanese version of the 17-item Hamilton Rating Scale for Depression (HAM-D) was used, with the severity of depression classified according to the report by Zimmerman et al. [13].

Treatment

Patients with *gas-predominant IBS *were treated by a team of physicians specializing in psychosomatic medicine, gastroenterology, and internal medicine. They were treated with antidepressants (amitriptyline and/or paroxetine) regardless of the presence or absence of a comorbid depressive disorder. They received only the standard dietary advice to avoid trigger foods. None were put on dietary interventions such as a low FODMAP (Fermentable Oligo-, Di-, Mono-saccharides, And Polyols), high fiber, or gluten-free diet during the treatment period.

After explaining the treatment protocol and the significance of antidepressant administration, informed consent was obtained from the patient or both the patient and a parent if the patient was 18 years of age or younger.

To avoid the gastrointestinal side effects caused by paroxetine, the patients were initially started on 10 mg/day of amitriptyline: our clinical experience is that most patients who have taken a certain amount of amitriptyline prior to taking selective serotonin reuptake inhibitors (SSRIs) experience few gastrointestinal side effects caused by SSRIs (unpublished data).

Amitriptyline was gradually increased to a maximum dose of 30 to 60 mg/day. If it caused side effects, such as obvious lightheadedness, severe dry mouth, or severe constipation, paroxetine was started as the main antidepressant, with the dose of amitriptyline gradually reduced while paroxetine was increased gradually from 10 to 20, 30, or 40 mg/day in response to clinical symptoms and side effects.

In addition, for all patients, other medications were given to control symptoms: 300 to 600 mg/day of trimebutine and/or probiotics were administered in combination with amitriptyline or paroxetine. Ramosetron (2.5 µg/day for women and 5.0 µg/day for men) or cholestyramine (1,000 to 2,000 mg/day) was administered as required to ameliorate diarrhea, either individually or in combination. If necessary, 10 mg/day of elobixibat or 250 to 1,500 mg/day of magnesium oxide was administered to relieve constipation. Thirty mg/day of tiquizium was administered as needed to relieve abdominal pain. The above peripheral and symptomatic medications were reduced or discontinued according to the degree of improvement in symptoms.

Assessment of the therapeutic effect of medication for IBS symptoms

We provisionally developed a prototype 11-point IBS Numerical Rating Scale (IBS-NRS) because there was no simple rating scale to assess adequately the therapeutic effect of medication on the following six IBS symptoms: abdominal bloating, ‘rumbling’, ‘flatulence’, abdominal pain, constipation, and diarrhea.

Patients were asked about their symptoms every two or three weeks and then asked to rate each IBS symptom from 0 to 10 (0=none; 10=worst imaginable) on our IBS-NRS scale. For abdominal pain: “How severe is your abdominal pain?” For bloating, ‘rumbling’, or ‘flatulence’: “How severe is your abdominal bloating, ‘rumbling’ or ‘flatulence’?” For constipation, diarrhea, or both: “How much have your bowel habits interfered with your life in the past two weeks?” The wording of the questions about each symptom in the IBS-NRS was largely taken directly from the IBS-SSS [11].

In this paper, ‘remission-like improvement’ was defined as achieving an IBS-NRS score of 3 or less for all six of the IBS symptoms listed above within 24 months after starting treatment and continuing for at least three months after achieving ‘remission-like improvement’ with no disturbance of usual daily or social life. Moderate improvement was defined as achieving an IBS-NRS score of 4 or 5 for all six IBS symptoms within 24 months. The patients included in this study were those who had ‘remission-like improvement’ within 24 months after the start of treatment and could be followed for at least six months and patients who were treated for at least 24 months without having ‘remission-like improvement’. Relapse was defined as a worsening of symptoms, accompanied by difficulty in their usual daily life or social activities and an IBS-NRS score of more than 3 for any IBS symptom after having previously achieved ‘remission-like improvement’.

Statistical analysis

Proportion, mean with standard deviation (SD), and median with range were used as descriptive statistics. The Fisher’s exact test was used for between-group comparison of categorical variables, and the Mann-Whitney U test and Kruskal-Wallis test were used for between-group comparison of continuous variables. All statistical analyses were performed using StatView ver 5.0 (HULINKS, Tokyo, Japan).

Ethical statement

This study was approved by the Ethics Committee of the BOOCS Clinic Fukuoka (Approval Number: 240118-1) and was implemented in compliance with the Declaration of Helsinki.

## Results

All 146 patients diagnosed as having IBS were classified based on a self-rating of which symptoms were worst and most bothersome. The number of patients in each subgroup, classified on the basis of symptoms rated as worst and most bothersome, is shown in Table 1.

**Table 1:** Characteristics of 146 consecutive patients diagnosed as having irritable bowel syndrome (IBS) according to the Rome IV criteria ^a^The subgroup of IBS for which the most bothersome symptom is abdominal pain. ^b^The subgroup of IBS for which the most bothersome symptom is both abdominal pain and diarrhea. ^c^The subgroup of IBS for which the most bothersome symptom is both abdominal pain and constipation ^d^The subgroup of IBS for which the most bothersome symptom is threatening or frequent diarrhea. ^e^The subgroup of IBS for which the most bothersome symptom is constipation. ^f^The subgroup of IBS for which the most bothersome symptom is abdominal bloating, distension, ‘flatulence’, or ‘rumbling’.

Subgroups classified according to the symptom rated as being the worst	Number of patients	Sex Female (%)	Age (years) Median (range)
*Pain-predominant IBS*^a^	51	31 (60.8)	20 (11-70)
*Pain & diarrhea-predominant IBS*^b^	52	33 (63.5)	30 (12-60)
*Pain & constipation-predominant IBS*^c^	1	1 (100.0)	38
*Diarrhea-predominant IBS*^d^	10	8 (80.0)	30 (21-50)
*Constipation-predominant IBS*^e^	1	1 (100.0)	32
*Gas-predominant IBS* ^f^	31	28 (90.3)	22 (14-43)
Total	146	102 (69.9)	24 (11-70)

Of the 146 IBS patients, 31 (21.2%) were classified into the *gas-predominant IBS *group. As compared with the other subgroups combined (n=115), the *gas-predominant IBS* group had a substantially higher proportion of female patients (90.3% versus 64.3%, P=0.004) and tended to be younger (median age 22 versus 25 years, P=0.098).

Characteristics of patients with *gas-predominant IBS*

Of the 31 patients with *gas-predominant IBS*, the data of 10 were excluded because they did not meet the required conditions regarding treatment with antidepressants as described above. The reasons for exclusion were the change of treatment center (n=4), inability to follow up for more than six months after achieving a ‘remission-like improvement’ (n=2), discontinuation of medication for more than two weeks due to personal reasons (n=3), and switching from paroxetine to escitalopram due to weight gain (n=1). Consequently, data from the remaining 21 patients, aged 14 to 30 years with *gas-predominant IBS*, were available for the study.

**Figure 1:**
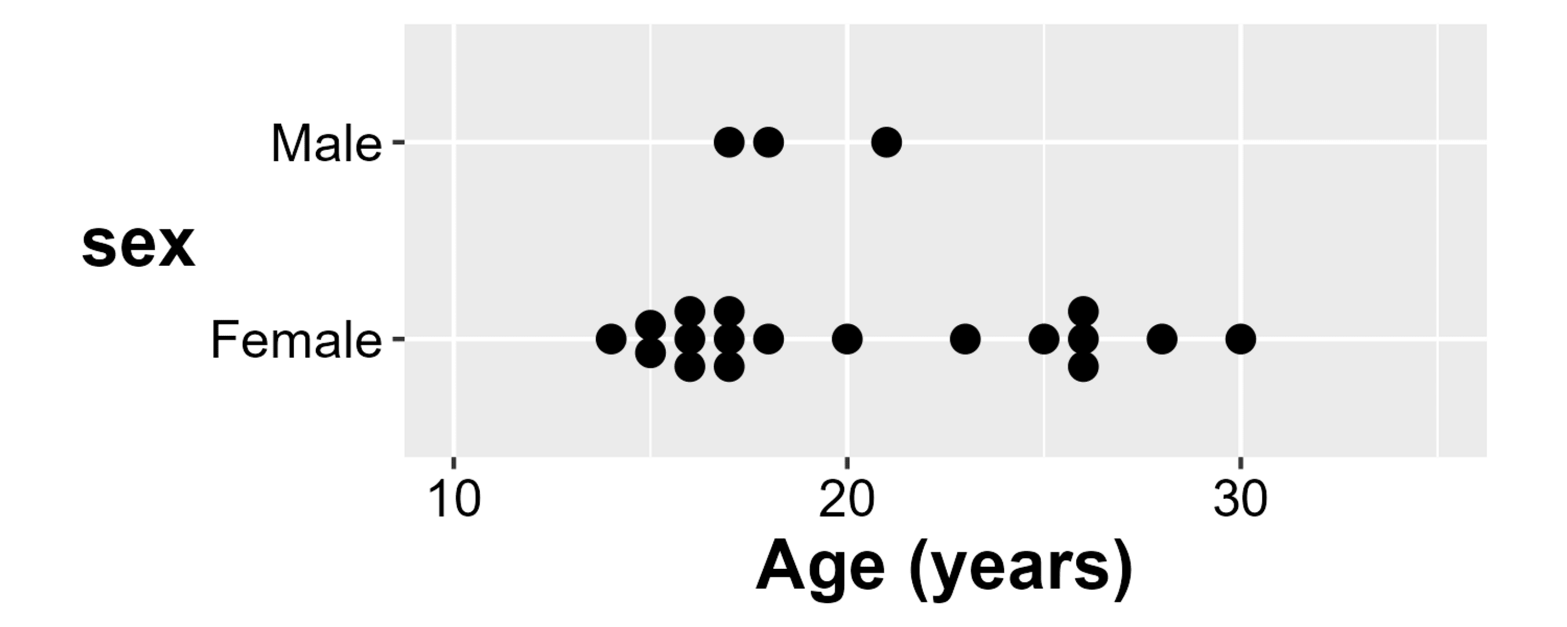
Clinical characteristics of 21 consecutive patients with predominant intestinal-gas symptom IBS (gas-predominant IBS): Sex and age To protect patient anonymity, information regarding the sex and age of these patients was removed from Tables 2, 5 and included in Figure 1 instead.

A profile of the 21 patients with *gas-predominant IBS *is shown in Figure 1 and Table 2. Eighty-six percent were female patients and more than half were under 20 years of age.

**Table 2:** Clinical characteristics of 21 consecutive patients with predominant intestinal-gas symptom IBS (gas-predominant IBS) HAM-D: Hamilton rating Scale for Depression; IBS: Irritable Bowel Syndrome; MDD: Major Depressive Disorder; STAI: State-Trait Anxiety Inventory ^a^The length of time that patients suffered from significant IBS symptoms is given in years, rounded to one decimal point or less. ^b^The subtype of IBS was determined according to the predominant disorder in bowel habits (ROME IV criteria). ^c^The severity of each intestinal-gas symptom was rated using an IBS-NRS scale and ranked in order of severity as follows: none, not at all; mild, not much; moderate, quite a lot; severe, completely. ^d^Rumbling is defined as rumbling in the bowel. ^e^Flatulence is defined as ‘excess flatulence expelled through the anus’ or ‘incontinence of flatulence’. ^f^The STAI questionnaire is composed of two scales: one to assess anxiety state and the other to assess trait. State anxiety is defined as a subjective feeling of nervousness. Trait anxiety is an individual’s underlying tendency to perceive a situation a certain way. The results were analyzed in five categories of anxiety as a state and as a trait. For women, the values obtained for the state anxiety scales range from 20 to 80 points, with the 20–21 range described as a very low level of anxiety, 22–30 as a low level of anxiety, 31–41 as moderate anxiety, 42–50 as high anxiety, and 51–80 as extremely high anxiety, and the values obtained for the trait anxiety scales range from 20 to 80 points, with the 20–23 range described as a very low level of anxiety, 24–33 as a low level of anxiety, 34–44 as moderate anxiety, 45–54 as high anxiety, and 55–80 as extremely high anxiety (The values of the five categories for men are omitted). ^g^Psychiatric diagnosis was determined according to DSM-V.

Case	Suffering period (years)^a^	Subtype of IBS^b^	Severity ranking of intestinal-gas symptoms^c^			Anxiety level (Score of STAI)^f^		Psychiatric diagnosis^g^
		(IBS-SSS score)	Bloating	Rumbling^d^	Flatulence^e^	State	Trait	(HAM-D score)
No. 1	11	Mixed type (360)	2^nd^ worst	3^rd^ worst	worst	High (45)	High (51)	None
No. 2	1	Constipation type (398)	2^nd^ worst	3^rd^ worst	worst	Extremely high (58)	Extremely high (62)	None
No. 3	4	Constipation type (395)	2^nd^ worst	2^nd^ worst	worst	Extremely high (51)	Extremely high (63)	None
No. 4	13	Mixed type (380)	2^nd^ worst	None	worst	High (47)	Extremely high (61)	None
No. 5	5	Diarrhea type (383)	2^nd^ worst	2^nd^ worst	worst	Extremely high (56)	Extremely high (55)	None
No. 6	1	Constipation type (381)	2^nd^ worst	None	worst	High (44)	High (48)	None
No. 7	2	Constipation type (391)	2^nd^ worst	2^nd^ worst	worst	Extremely high (69)	Extremely high (70)	None
No. 8	4	Diarrhea type (410)	2^nd^ worst	3^rd^ worst	worst	Extremely high (58)	Extremely high (62)	None
No. 9	2	Constipation type (359)	2^nd^ worst	2^nd^ worst	worst	Extremely high (60)	Extremely high (62)	None
No. 10	11	Constipation type (364)	2^nd^ worst	worst	worst	Extremely high (60)	Extremely high (62)	None
No. 11	3	Constipation type (320)	worst	3^rd^ worst	2^nd^ worst	Extremely high (72)	Extremely high (54)	None
No. 12	20	Diarrhea type (393)	2^nd^ worst	2^nd^ worst	worst	Extremely high (68)	Extremely high (71)	MDD (19)
No. 13	20	Mixed type (433)	2^nd^ worst	2^nd^ worst	worst	Extremely high (75)	Extremely high (74)	MDD (17)
No. 14	2	Diarrhea type (406)	3^rd^ worst	worst	worst	Extremely high (58)	Extremely high (64)	None
No. 15	2	Mixed type (362)	2^nd^ worst	worst	2^nd^ worst	High (44)	Extremely high (59)	None
No. 16	2	Diarrhea type (367)	3^rd^ worst	worst	worst	Moderate (35)	High (53)	None
No. 17	15	Diarrhea type (387)	2^nd^ worst	None	worst	High (44)	Extremely high (55)	None
No. 18	2	Diarrhea type (422)	2^nd^ worst	worst	None	Extremely high (66)	Extremely high (68)	None
No. 19	1	Mixed type (320)	None	2^nd^ worst	worst	Extremely high (53)	Extremely high (60)	None
No. 20	3	Diarrhea type (357)	2^nd^ worst	2^nd^ worst	worst	Extremely high (57)	Extremely high (51)	None
No. 21	10	Diarrhea type (372)	None	worst	2^nd^ worst	Extremely high (62)	Extremely high (69)	None

All of the patients had considerable difficulty in their usual social life, with their quality of life being severely impaired due to their IBS symptoms, especially intestinal-gas. More than a third of this group, eight patients, had continuously suffered from severe intestinal-gas and other IBS symptoms for more than 10 years. All patients had previously visited several hospitals and had been treated with conventional medications, such as drugs to reduce symptoms that have peripheral action or with anxiolytics, but without antidepressants. Treatment had been done in the internal medicine, pediatrics, psychiatry, or gastroenterology departments of other hospitals, but the patients had not improved and were considered to be refractory to treatment.

All of the patients had a severity score of more than 300 (mean±SD: 379±29) and were classified as having severe IBS using the IBS-SSS questionnaire (Table 3).

**Table 3:** Most bothersome intestinal-gas symptom of 21 consecutive patients with predominant intestinal-gas symptom IBS (gas-predominant IBS) IBS-SSS: Irritable Bowel Syndrome Severity Scoring System; MDD: Major Depressive Disorder; STAI: State-Trait Anxiety Inventory ^a^The length of time that patients suffered from significant IBS symptoms is given in years, rounded to one decimal point or less. ^b^The severity of IBS of each patient with *gas-predominant IBS* was assessed using the IBS-SSS questionnaire. ^c^Psychiatric diagnosis was determined according to DSM-V. ^d^The STAI questionnaire is composed of two scales: one to assess anxiety state and the other to assess trait. State anxiety is defined as a subjective feeling of nervousness. Trait anxiety is an individual’s underlying tendency to perceive a situation a certain way. For women, the values obtained for the trait anxiety scale range from 20 to 80 points, with the 20–21 range described as a very low level of anxiety, 22-31 as a low level of anxiety, 31–41 as moderate anxiety, 42–50 as high anxiety, and 51-80 as extremely high anxiety. (The values of the five categories for men are omitted) ^e^‘Flatulence’ is defined as ‘excess flatulence expelled through the anus’ or ‘incontinence of flatulence’. ^f^‘Rumbling’ is defined as rumbling in the bowel. ^g^‘Flatulence’ and ‘rumbling’ are equally severe symptoms. ^h^Their severity of depression was evaluated as being moderate based on their Hamilton Rating Scale for Depression (HAM-D) scores according to the definition of Zimmerman et al. [13].

Most bothersome intestinal-gas symptom	Number of patients (%)	Suffering period (years)^a^	IBS-SSS^b^	Psychiatric diagnosis^c^	Anxiety level (STAI Score)^d^	
					State	Trait
‘Flatulence’^e^	14 (66.7%)	7.3±7.1	382±27	MDD: 2 cases^h^	55.6±9.8	60.8±7.4
‘Rumbling’^f^	3 (14.3%)	4.7±4.6	385±32	None	57.3±11.7	65.3±5.5
‘Flatulence’ and ‘rumbling’^g^	3 (14.3%)	5.0±5.2	379±23	None	51.0±13.9	59.7±5.9
Bloating	1 (4.7%)	3	320	None	72	54
Total	21 (100.0%)	6.4±6.3	379±29	MDD: 2 cases^h^	56.0±10.6	61.0±6.9

As shown in Tables 2, 3, the most bothersome symptoms were ‘flatulence’ in 14 (66.7%), ‘rumbling’ in three (14.3%), both ‘flatulence’ and ‘rumbling’ in three (14.3%) and bloating in one (4.8%). Notably, 17 (81.0%) of the 21 patients rated ‘flatulence’ and six (28.6%) rated ‘rumbling’ as one of the worst symptoms (Table 3). No statistically significant differences were found according to the categories of the most bothersome intestinal-gas symptom with respect to the suffering period (P=0.92), IBS-SSS (P=0.48), state anxiety (P=0.47), and trait anxiety (P=0.34).

Of the 21 patients, two (9.5%) had a diagnosis of comorbid major depressive disorder, with HAM-D scores of 17 and 19, respectively. Their severity of depression was evaluated as being moderate based on their HAM-D scores. The other 19 (90.5%) patients had no comorbid psychiatric disorder such as depressive or anxiety disorder. However, the STAI showed very high anxiety scores for both state and trait for 15 (71.4%) of these patients.

Effect of treatment with antidepressants

As shown in Tables 4, 5, 18 (85.7%) of the 21 patients in the *gas-predominant IBS *group achieved ‘remission-like improvement’, including two who achieved ‘remission-like improvement’ when treatment was done with 50 to 60 mg/day of amitriptyline only.

**Table 4:** Effect of treatment with paroxetine or amitriptyline for 21 consecutive patients with predominant intestinal-gas symptom IBS (gas-predominant IBS) M: months; N/A: not applicable ^a^‘Remission-like improvement’ was defined as achieving an IBS-NRS of 3 or less for all six IBS symptoms within 24 months after starting treatment, with the improvement lasting at least three months with no disturbance to daily or social life. Moderate improvement was defined as achieving an IBS-NRS of 4 or 5 for all six IBS symptoms within 24 months. ^b^Medication status at the time of ‘remission-like improvement’ and at the end of follow-up for patients with moderate improvement. ^c^The length of the treatment period required to achieve ‘remission-like improvement’ is given in months, rounded to one decimal point or less. ^d^Relapse was defined as a worsening of the symptoms of IBS patients accompanied by difficulty in usual daily life or social activities and an IBS-NRS score of more than 3 for any IBS symptom after achieving ‘remission-like improvement’. All patients who achieved ‘remission-like improvement’ were followed for at least six months.

Therapeutic effect^a^	Number of patients (%)	Medication (N)^b^	Duration (M) of treatment to achieve ‘remission-like improvement’^c^	Relapse^d^
‘Remission-like improvement’	18 (85.7%)	Paroxetine (14), Amitriptyline (2), Paroxetine + Amitriptyline (2)	10.2±5.3	None
Moderate improvement	3 (14.3%)	Paroxetine (1), Amitriptyline (2)	N/A	N/A
No change	0 (0.0%)	N/A	N/A	N/A
Aggravation	0 (0.0%)	N/A	N/A	N/A
Total	21 (100.0%)	Paroxetine (15), Amitriptyline (4), Paroxetine + Amitriptyline (2)	N/A	N/A

**Table 5:** Effect of treatment with paroxetine or amitriptyline for 21 consecutive patients with predominant intestinal-gas symptom IBS (gas-predominant IBS) IBS-NRS: Irritable Bowel Syndrome-Numerical Rating Scale; M: months; N/A: not applicable ^a^‘Remission-like improvement’ was defined as achieving an IBS-NRS score of 3 or less for all six IBS symptoms within 24 months after starting treatment, with the improvement lasting at least six months with no disturbance to daily or social life. Moderate improvement was defined as achieving an IBS-NRS score of 4 or 5 for all six IBS symptoms within 24 months. ^b^The length of the treatment period required to achieve ‘remission-like improvement’ is given in months, rounded to one decimal point or less. ^c^Every two or three weeks, patients were asked to rate six symptoms (bloating, flatulence, rumbling, abdominal pain, diarrhea and constipation) on an IBS-NRS scale from 0 to 10 (0=absence of symptom; 10=highest expression of the symptom). The IBS-NRS score for the most bothersome symptom is indicated. ^d^IBS-NRS score at the time of achieving ‘remission-like improvement’.

Case	Medication (mg/day)	Therapeutic effect^a^	Duration of treatment to achieve ‘remission-like improvement’^b^	IBS-NRS score^c^ of the most bothersome symptom after starting treatment			Relapse within six months after achieving ‘remission-like improvement’
				Most bothersome IBS symptom	After 6M	After 12M	
No. 1	Paroxetine (40)	Remission-like improvement	8M	Flatulence	4	1	None
No. 2	Paroxetine (40)	Remission-like improvement	7M	Flatulence	5	0	None
No. 3	Paroxetine (40)	Remission-like improvement	9M	Flatulence	5	2	None
No. 4	Paroxetine (40)	Remission-like improvement	5M	Flatulence	3	3	None
No. 5	Paroxetine (40)	Remission-like improvement	14M (2)^d^	Flatulence	6	4	None
No. 6	Amitriptyline (50)	Remission-like improvement	5M	Flatulence	3	1	None
No. 7	Paroxetine (30)	Remission-like improvement	8M	Flatulence	4	0	None
No. 8	Paroxetine (40)	Remission-like improvement	18M (3)^d^	Flatulence	7	5	None
No. 9	Paroxetine (40)	Remission-like improvement	13M (2)^d^	Flatulence	6	4	None
								No. 10	Paroxetine (40)	Remission-like improvement	20M (2)^d^	Rumbling	7	5	None
Flatulence															
No. 11	Amitriptyline (60)	Remission-like improvement	23M (3)^d^	Bloating	6	5	None
No. 12	Paroxetine (40)	Moderate improvement	N/A	Flatulence	9	7 (After 24M: 5)	N/A
No. 13	Amitriptyline (60)	Moderate improvement	N/A	Flatulence	6	5 (After 24M: 4)	N/A
No. 14	Paroxetine (40)	Remission-like improvement	8M	Rumbling	4	1	None
				Flatulence			
No. 15	Paroxetine (30)	Remission-like improvement	17M (1)^d^	Rumbling	6	5	None
No. 16	Amitriptyline (20) & Paroxetine (20)	Remission-like improvement	5M	Rumbling, Flatulence	2	1	None
No. 17	Amitriptyline (50)	Moderate improvement	N/A	Flatulence	7	6 (After 24M: 4)	N/A
No. 18	Paroxetine (40)	Remission-like improvement	7M	Rumbling	4	1	None
No. 19	Paroxetine (40)	Remission-like improvement	6M	Flatulence	3	1	None
No. 20	Paroxetine (40)	Remission-like improvement	6M	Flatulence	3	2	None
No. 21	Amitriptyline (10) & Paroxetine (30)	Remission-like improvement	10M	Rumbling	5	2	None

Sixteen of the 18 patients who achieved ‘remission-like improvement’ were initially treated with amitriptyline, but the dose had to be reduced and paroxetine added or amitriptyline was tapered off and switched to paroxetine because of side effects, such as orthostatic dizziness and severe dry mouth which were observed at 30 to 60 mg/day. Of these 16 patients, fourteen were being administered paroxetine (30 to 40 mg/day) only at the time they achieved ‘remission-like improvement’. The remaining two patients were being administered paroxetine and amitriptyline in combination at the time they achieved ‘remission-like improvement’.

None of the 17 patients who were treated with amitriptyline before receiving paroxetine complained of gastrointestinal side effects caused by paroxetine throughout treatment, regardless of whether or not they were with or without ‘remission-like improvement’.

**Figure 2:**
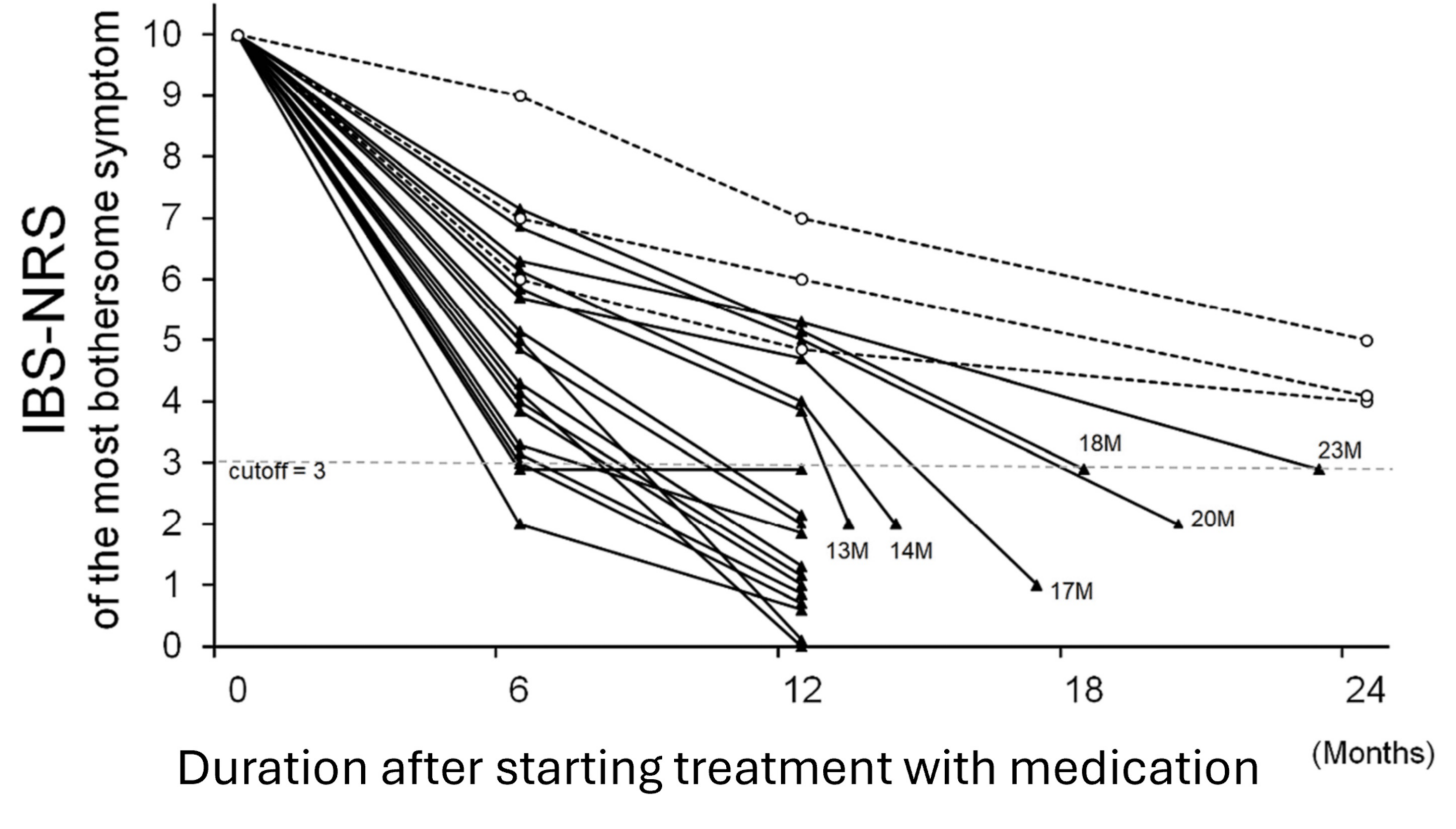
Effect of medication on the IBS-NRS score for the most bothersome IBS symptom after starting treatment for 21 consecutive patients with intestinal-gas symptom predominant IBS (gas-predominant IBS) IBS-NRS: Irritable Bowel Syndrome-Numeric Rating Scale The most bothersome gas symptom of IBS was ‘flatulence’, ‘rumbling’, both ‘flatulence’ and ‘rumbling’, or bloating. Patients rated the symptom on an IBS-NRS scale from 0 to 10 (0=absence of symptom; 10=highest expression of the symptom). Solid lines represent 18 cases with ‘remission-like improvement’, dashed lines represent three cases with moderate improvement. For cases that took more than 12 months from the start of treatment to achieve ‘remission-like improvement’, the duration (months) of treatment to achieve ‘remission-like improvement’ is noted.

The duration of treatment required to achieve ‘remission-like improvement’ was relatively long, ranging from five to 23 months. The IBS-NRS scores for abdominal pain, diarrhea, and constipation of all 18 patients who achieved ‘remission-like improvement’ fell below 3 significantly earlier than those for ‘flatulence’, ‘rumbling’, or bloating (data not shown). As a result, the most bothersome symptom remained the same as before treatment for all patients throughout the treatment period. Changes in the IBS-NRS scores for the most bothersome symptom, i.e. ‘flatulence’, ‘rumbling’, or bloating, during treatment, are shown in Figure 2 and Table 4.

Of the 18 patients who achieved ‘remission-like improvement’, 13 were able to stop all of their medications, and none of the 18 patients had a relapse within the six months after achieving ‘remission-like improvement’. The three patients who did not achieve ‘remission-like improvement’ within 24 months had IBS-NRS scores for ‘flatulence’ greater than 4, even though the IBS-NRS scores for abdominal pain, diarrhea, or constipation had fallen below 3 much earlier.

## Discussion

In the present study, we have shown that there is a distinct group of Japanese IBS patients who complain of intestinal-gas symptoms such as ‘flatulence’ or ‘rumbling’ as their most distressing symptom.

In our *gas-predominant IBS* group, ‘flatulence’ was most often rated as the most bothersome symptom and ‘rumbling’ as the second most common. The most notable feature of *gas-predominant IBS* shown in the present study was that the disturbance of daily and social life due to ‘flatulence’ or ‘rumbling’ was extremely serious, causing significant impairment of quality of life and loss of productivity at work or school. Our *gas-predominant IBS* group had been treatment-refractory, and none of the studied patients had been successfully treated at other hospitals that used the conventional medications for IBS.

Intestinal-gas symptoms, notably bloating, are common for IBS patients [2,4]. Lembo et al. [4] in a study of 443 consecutive patients with IBS found that bloating was listed by 60% as their most bothersome symptom. Thus, intestinal-gas is an important, distressful matter for IBS patients. However, there is comparatively little research into the IBS patients who complain of intestinal-gas as a predominant symptom, probably because it is not specific enough for the diagnosis of IBS.

Most previous studies of intestinal-gas in IBS have focused on bloating, not on ‘flatulence’ or ‘rumbling’ [2,3]. Only a few studies of the ‘flatulence’ of patients with IBS have strictly defined it as ‘excessive flatulence expelled through the anus’ or ‘incontinence of flatulence’ [10] as was done in this paper. Despite the fact that a significant proportion of people with IBS suffer from ‘flatulence’ and/or ‘rumbling’, the importance of these symptoms in IBS has not been seriously discussed. Maxton et al. [5] suggested the importance of ‘flatulence’ (written as ‘excess wind’ in their paper) in IBS patients. They reported that among 100 patients with IBS, 66 had a high ‘flatulence’ severity score, eight rated ‘flatulence’ as being the worst symptom, and nine had it as the second worst. Recently, Duracinsky et al. [9] suggested that ‘flatulence’, ‘rumbling’ (written as stomach rumbling in their paper), and difficult gas evacuation as well as bloating were gas symptoms comparatively specific to IBS. The Intestinal Gas Questionnaire they developed was used with psychometric validation to investigate patients recruited from three countries (UK, Spain, and France).

Fishbain et al. [14] reported the case of a male white American, diagnosed with IBS by a gastroenterologist, who was severely bothered by incontinence of malodorous ‘flatulence’. This is the only case report we could find of an IBS patient who suffered most from ‘flatulence’.

In the present study, most patients who complained of ‘flatulence’ as their most distressing symptom reported that they became very nervous and intensely anxious in crowded places, such as at school or their office, because their ‘flatulence’ in public caused strong feelings of embarrassment. The fact that ‘flatulence’ is often accompanied by foul odors and unusual sounds exacerbates embarrassment. The emotional tension caused by anticipatory anxiety in public aggravates ‘flatulence’ or ‘rumbling’, creating a vicious cycle. As a result, IBS patients with predominantly intestinal-gas symptoms, especially those with ‘flatulence’ or ‘rumbling’, tend to avoid as much as possible going to places where people congregate. This causes significant restrictions in their daily life and social activities, and they get very tired psychologically.

All of the subjects in this study were Japanese, and ‘flatulence’ in public, the focus of this study, is one of the most embarrassing and distressing situations imaginable for so many Japanese people, and specific cultural aspects of Japan, notably the concept of shame, may well be associated with the impaired quality of life in the gas-predominant group of patients with IBS of this study. It has often been suggested that Japanese culture has a foundation in feelings of shame, and the idea that Japan has a culture of shame has been widely discussed [15,16]. It may be possible to say that Japan’s culture of shame may contribute to the exacerbation of *gas-predominant IBS*, with the most bothersome symptoms being ‘flatulence’ or ‘rumbling’. However, the existence of a *gas-predominant IBS* group for which the most bothersome symptoms are ‘flatulence’, or ‘rumbling’ may not be specific to the Japanese population. Because, for many people in any country, ‘flatulence’ and loud ‘rumbling’ in public can be extremely embarrassing, and several previous reports [5,14] suggest the existence of IBS patients in other countries who are most bothered by ‘flatulence’ or ‘rumbling’.

Many of the IBS patients in this study had not been able to talk about their ‘flatulence’ problems because of embarrassment and shame, even to their families as well as the doctors. It is possible that IBS patients in any country also do not clearly communicate their ‘flatulence’ problems to their doctors because they are too embarrassed, resulting in the doctors not being able to understand the exact condition of their IBS patients. The regional differences in the prevalence of *gas-predominant IBS* warrant further investigation. In the future, multicenter or international studies of the *gas-predominant IBS* subgroup in countries around the world may be desirable, especially from the point of view of cultural variables.

Previous epidemiological studies have shown that more women are affected by IBS than men [17]. This review also concluded that notable bloating and/or abdominal distension are more common and severe for women than for men. Similarly, the present study suggests that *gas-predominant IBS* is more prevalent in women than in men. Notably, more than half of the patients with refractory *gas-predominant IBS* were under 20 years of age. This finding suggests that adolescence, a crucial period for social development, may play a role in exacerbating symptoms of ‘flatulence’ and ‘rumbling’. Adolescents are particularly sensitive to issues of peer acceptance, rejection, and general approval, which may contribute to the severity of these gas-related symptoms. In this paper, the proportion of patients with *gas-predominant IBS* was about 20% of all IBS patients, however, this may not reflect the actual incidence. Because many patients with refractory IBS attend our clinic, the proportion of *gas-predominant IBS* in this study may be higher than that of the general population of IBS patients in Japan.

Despite the increasing number of hopeful pharmacotherapies and dietary interventions, an effective management strategy for intestinal-gas that includes ‘flatulence’ or ‘rumbling’ has not been established, and intestinal-gas as a symptom of IBS remains extremely difficult to treat [6].　

For the assessment of the efficacy of pharmacotherapy for *gas-predominant IBS*, we provisionally defined ‘remission-like improvement’ in this paper as a high level of control, including the absence or minimization of IBS symptoms and the absence of exacerbations for more than half a year. A large proportion of our patients with *gas-predominant IBS* had ‘remission-like improvement’ when treatment was centered on antidepressants.

The peripheral and symptomatic medications, trimebutine, ramosetron, cholestyramine, magnesium oxide, elobixibat, tiquizium, or probiotics, were co-administered with antidepressants (amitriptyline, paroxetine, or both) according to the symptoms of each patient. However, these combined drugs only relieve the symptoms, thus the treatment effect is tentative. In addition, these co-administered medications were reduced or stopped before the patient achieved ‘remission-like improvement’; thus, the antidepressants may have played an important role in the marked improvement in disease status in all cases of patients who achieved ‘remission-like improvement’. The antidepressants amitriptyline [18,19] and paroxetine [20] have been indicated to be beneficial for the treatment of patients with IBS. Ford et al. [21] described in a systematic review and meta-analysis that tricyclic antidepressants and SSRIs are probably effective treatments for IBS. In addition, Moshiree et al. [3] recommended the use of central neuromodulators (e.g., antidepressants) to treat bloating and abdominal distention in their review of the management of abdominal bloating and distension. In the aforementioned case report, the authors indicate that treatment with the SSRI fluoxetine significantly improved the symptoms of the IBS patient, who had suffered most from flatulence and had been refractory to various treatments [14]. On the other hand, previous studies have shown conflicting efficacy of antidepressants for the intestinal-gas symptoms in patients with IBS, and most have focused on bloating, not on ‘flatulence’ or ‘rumbling’ [22,23].　

In this study, patients with *gas-predominant IBS* required a relatively long period of treatment with antidepressants to achieve ‘remission-like improvement’: six to 24 months is a longer period of treatment than that used in previous studies in which the efficacy of antidepressants for IBS patients was evaluated for 12 weeks or less [21]. Based on our results, it seems that the treatment of *gas-predominant IBS *with antidepressants should be intensified in terms of treatment duration compared to the treatment for IBS patients whose predominant symptoms are diarrhea, constipation, or abdominal pain. In addition, it is of interest that most of the patients who achieved ‘remission-like improvement’ in this study had no comorbid psychiatric disorder, such as depression or anxiety disorder. This finding is consistent with several studies on the efficacy of antidepressants on IBS that have shown no correlation between improvement in IBS symptoms and depression scores [22,24].　

Camilleri [25] stated that “pharmacotherapies have not been shown to alter the long-term or history of IBS”. Although the required period of ‘remission-like improvement’ lasting at least six months was not necessarily long in the patients who improved significantly with antidepressants in the present study, the fact that none had a relapse within six months of treatment suggests that intensified pharmacotherapy with an adequate dose of antidepressant medication for a sufficient treatment period can relieve the severe symptoms of patients with refractory IBS for a considerable period, and the fact that none of the patients who achieved ‘remission-like improvement’ had a relapse within the six months after treatment suggests that strengthened pharmacotherapy with an adequate dose of antidepressant for a sufficient treatment period can relieve the severe symptoms of patients with refractory *gas-predominant IBS* for a considerable period of time. More research is needed to see if intensified pharmacotherapy with antidepressants can alter the long-term or history of IBS.

The mechanism underlying the beneficial effect of paroxetine or amitriptyline on the intestinal-gas symptom of IBS patients is considered to be multifactorial: the precise mechanism is unclear. Antidepressants may affect both the central serotonergic nervous system and the enteric serotonergic system, which may be involved in the motility and perception of the bowel. In addition, it can be postulated that antidepressants improve intestinal-gas symptoms by affecting microbiota-gut-brain interactions [26]. The serotonergic anxiolytic effect of paroxetine may be involved to some extent in the improvement in IBS symptoms: many patients with *gas-predominant IBS* have high levels of anxiety.

In practice, gastrointestinal side effects with paroxetine are a very important issue in the treatment of IBS patients with serious gastrointestinal symptoms, as shown in our previous [27] and other studies [28]. This is because side effects such as nausea or stomach discomfort caused by SSRIs make IBS patients feel as if their IBS is getting worse. Interestingly, our clinical experience in the present study is that the administration of amitriptyline for a period of time prior to paroxetine administration prevents or considerably reduces the occurrence of gastrointestinal side effects caused by paroxetine, which may have resulted from desensitization of both central and enteric serotonergic receptors by administration of amitriptyline for a sufficient period of time, with a weaker inhibiting property of serotonin transporter compared to SSRIs. This suggests that administration of amitriptyline at low doses that do not cause obvious adverse effects for a period of time prior to treatment with the SSRI paroxetine may be an effective strategy to prevent the occurrence of SSRI-induced gastrointestinal side effects leading to discontinuation of IBS treatment. In addition, when using paroxetine in clinical practice the discontinuation syndrome related to the withdrawal of the SSRI paroxetine, such as characteristic dizziness or nausea, should be taken into account with great caution in order to avoid patients dropping out of treatment, as we [29] and others [28] have previously shown.

This study has several limitations. First, it is a preliminary study with a small sample size done with only Japanese patients with *gas-predominant IBS* who complained of ‘flatulence’, ‘rumbling’, or bloating as their most distressing symptom. In addition, there is a potential selection bias from a single center. Further studies are needed on the prevalence and regional specificity of *gas-predominant IBS* in Japan and other countries. Second, the pharmacotherapy trial in this study, which was designed to test the efficacy of antidepressants in patients with *gas-predominant IBS*, was an open-label, no-control trial that lacked randomization and blinding. The type, dosage, and duration of medications to treat symptoms that were administered in combination with antidepressants were different for each patient. Given the findings of our study, future investigations will be needed to focus on the most appropriate use of paroxetine for the treatment of *gas-predominant IBS*. Large-scale, double-blind, placebo-controlled trials should be conducted in order to elucidate the optimal dose and duration of treatment with paroxetine. Third, this study used a prototype IBS-NRS to assess the subjective symptoms of IBS. However, this was not validated, and the IBS-NRS should be validated in a future study.

## Conclusions

In conclusion, our study results support our clinical impression that there exists a distinct intestinal-gas symptom predominant subgroup of patients with IBS *(gas-predominant IBS)* for whom the most distressing symptoms are flatulence or rumbling. Paroxetine administration for a relatively long period shows promising results requiring further validation in the treatment of refractory *gas-predominant IBS*.
